# 24-Hour Measurement of Gastric pH in Rural South Africa

**DOI:** 10.1155/2015/658106

**Published:** 2015-03-12

**Authors:** Alastair M. Sammon, Eugene J. Ndebia, Ekambaram Umapathy, Jehu E. Iputo

**Affiliations:** Department of Physiology, School of Medicine, Walter Sisulu University, Private Bag X1, Mthatha, Eastern Cape 5100, South Africa

## Abstract

*Background*. Previous studies have established norms of 24-hour gastric pH profiles for western countries. This study was designed to establish the pattern for a rural African population with a high incidence of oesophageal cancer. *Methods*. After lower oesophageal manometry a probe was placed 10 cm distal to the lower oesophageal sphincter. We carried out 24-hour ambulatory monitoring of gastric pH on 59 healthy subjects. This was satisfactorily completed on 26 female and 18 male (age 21–64, median 35) subjects in the Transkei region of South Africa. *Results*. The mean 24 hour gastric pH was 2.84 and the mean night-time pH was 3.7. 40 volunteers recorded a night-time pH reaching over 4. 33 volunteers recorded a night-time pH over 7. Night-time alkalinisation was present for 136.4 minutes (25th centile 22.8, 75th centile 208.1) at pH4 or over, and 79.3 (2.5, 122.7) minutes at pH7 or over. Episodes of rapid alkaline rise were 17 (10, 47). 21.1% of these occurred while supine. 35 of 36 tested subjects were positive for *H. pylori* IgG. *Conclusion*. Gastric alkalinisation is common in Transkei, at a higher pH than that reported in other studies, and is sustained longer. Nighttime alkalinisation is frequent. This suggests a high level of duodenogastric reflux.

## 1. Introduction

Transkei, part of the Eastern Cape of South Africa, is one of the three areas of the world with a very high reported incidence of squamous cancer of the oesophagus [1, 2]. There is as yet no universally accepted mechanism of causation in these areas, though many theories have been put forward [3–7].

We have demonstrated in previous studies that the upper gastrointestinal tract in Transkei differs in some indices from published norms [8–10], and these differences may be related to oesophageal carcinogenesis. This study was designed to establish the prevalent pattern of gastric pH over 24 hours within this community.

## 2. Methods

We obtained permission for the study from the Research Committee of Walter Sisulu University (reference 00011A-04). We recruited volunteers from healthy adult inhabitants of the area surrounding Canzibe Hospital in the rural Transkei region of the Eastern Cape. They were selected using a self-exclusion questionnaire and not based on diet, education, or income. Those with upper gastrointestinal complaints were eliminated. Healthy volunteers aged between 18 and 65 years with no past medical history of digestive disease or associated symptoms, diabetes, or neurological disease were recruited. None were consuming drugs at the time of the investigation.

We recorded height and weight and calculated BMI. We measured gastric pH using an antimony electrode. The lower oesophageal sphincter was defined using a pressure transducer (Gaeltec, model ICT/B) using the station pull through technique. The upper border of the sphincter was defined by a sudden increase of pressure compared to the esophageal body. Participants with no detectable LES with this technique were excluded from the study. The test was initiated after an overnight fast. After location of the lower oesophageal sphincter by manometry, the probe was passed and the electrode was positioned 10 cm below the sphincter. It was secured to the nose and face and connected to the recording device. We instructed the subject on how to record meal times and supine episodes, and the participant was then allowed to go home for the 24 hours of the study.

Gastric pH was sampled 50 times per second. Data was recorded in an Ohmega ambulatory pH-impedance recorder and downloaded to a computer at the end of the recording period. We carried out analysis visually and also using MMS software version 8.19 (Medical Measurements Systems B.V., Netherlands).

We excluded meal times from analysis. We defined a rapid alkaline rise as a rise in less than 10 seconds from a baseline of pH2 or less to a pH of 4 or more. Nighttime was taken as midnight to 6 am. We defined a nighttime pH of greater than 4 as nighttime alkalinisation and recorded the total lengths of time over pH4 and over pH7.

We measured rapid alkaline rises visually. Other measures were provided by the software analysis programme.

We measured* H. pylori* serology using* Helicobacter pylori* IgG Elisa Kit (EIA3484, DRG International Inc.). This was carried out as a secondary investigation on all volunteers who were then available (80% of volunteers).

We expressed nonparametric data as median, 25th and 75th centiles.

## 3. Results

59 subjects were studied. 15 records were unusable because of a recording of less than 23 hours (one), technical faults with the recording device (five), probe misplacement (one), and poorly recorded meal or supine periods (eight). Recording times of the remaining 45 were 24 hours (43 records), 23h53 and 23h30. 44 subjects had a minimum intragastric pH of 1.1 or less. One had a minimum pH of 4.0 and was eliminated from the study. 26 were female and 18 were male. Median BMI was 25.3 (range of 17.9 to 40.3). 35 of 36 subjects analysed for* H. pylori* IgG were positive.

**Figure 1:**
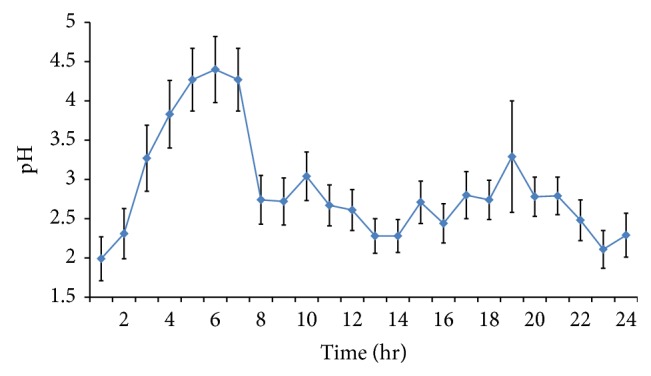
Aggregated 24 hr gastric pH (± one standard deviation) of 44 subjects (starting at midnight).

Figure 1 shows an aggregated hourly gastric pH for the 44 subjects. The mean 24-hour gastric pH was 2.84. The mean nighttime pH was 3.7 (see Table 1 and Figure 1).

**Figure 2:**
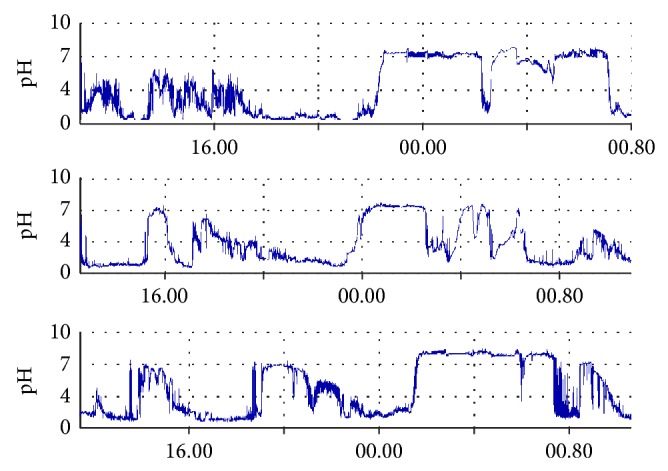
Samples of records with prolonged nighttime alkalinisation.

There were many episodes of nighttime alkalinisation (NTA) with plateaux of up to 9-hour duration. These were not related to food or liquid ingestion. 40 subjects recorded a nighttime pH reaching over 4 and 33 recorded a pH over 7. In 27 subjects, a nighttime pH of over 7 was present for over one hour and in 12 cases for over 2 hours (see Table 1 and Figure 2).

17 subjects achieved a pH of over 8, with a range of duration of 6 seconds up to 130 minutes. Figure 2 shows examples of nighttime alkaline plateaux. The median time in NTA Ph >7 for all 44 volunteers was 96.3 minutes. Including only those (33) who did have NTA >7, the median time was 107.1 minutes.

**Figure 3:**
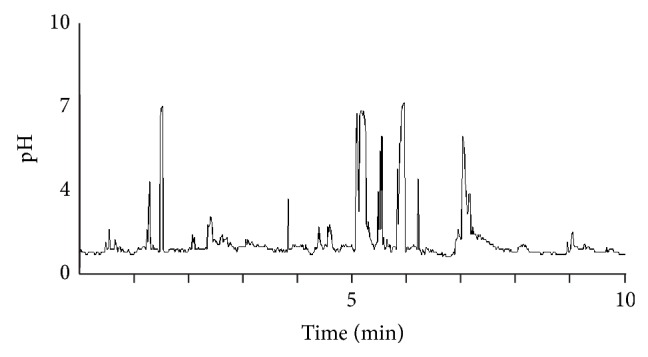
Sample of a record with rapid alkaline rises.

Rapid rises of gastric pH are as demonstrated in Figure 3 and the numbers are tabulated in Table 1. Occurrences were 17 (10, 47). 15.5% occurred in the first hour of recording. 21.1% occurred with the subject supine.

## 4. Discussion

Probe placement is critical, with different pH patterns evident in various zones of the stomach [11]. 10 cm distal to the lower oesophageal sphincter, as used in this study, is described as midgastric or in the corpus [12–14] and is a standard measuring position. This site is moderately influenced by antral fluid. The pH is reproducible over a 24-hour period [13]. Placement 5 cm distal to the lower oesophageal sphincter has been used in some studies [11, 15] and produces results less influenced by the antral content and distinct from midgastric results [11].

There were a relatively high number of unsatisfactory recordings. Reasons include the physical stress on the device and its cables being used in a rural setting where subjects must continue their daily living. Lack of familiarity with using electronic devices may account for the high number of failures to record meals and supine periods. While the study was successful, the number of recording failures underlines the difficulties of such a study in rural Africa. Several reported studies have included 35 or 36 volunteers [16–18]. Closure of this study at a total of 44 successful subjects provided data comparable with these studies.

We found a higher than usual mean gastric pH. The hourly gastric pH for the 24-hour period is continuously over 2. Other published graphs of composite or multiple subjects' hourly pH recordings show that it is mostly between pH1 and pH2 [13, 19]. Published representative graphs of single subjects [15, 20, 21] also show a baseline pH between 1 and 2. Studies with mean or median gastric pH are shown in Table 2 and include figures from pH1.3 to pH2.39 [16, 18, 22–24].

We found a prolonged nighttime rise in pH compared to other studies [13–15, 19, 20, 25], whose subjects showed brief periods of alkalinisation which were normally followed by a return to acidic pH. Published figures for total time at >pH4 are 8.8% to 20.03% [12, 16, 17, 22, 26] (see Table 2). Ours, at 22.2%, is higher than all but only a little higher than the last value. However Roman et al. [22] recorded a supine pH >4, 4.4% of the time, compared with 26% in our study. In summary, daytime pH and 24-hour profile are slightly raised compared with similar studies in North America/Europe. Nighttime and supine pH are more markedly raised and clearly different from other published series.

This study had two measures compatible with duodenogastric reflux (DGR). First, the presence of rapid rises in pH which were not associated with food intake. The rapid alkaline rises described have no adequate explanation apart from brief episodes of DGR. The occurrence of DGR is regarded as a normal event; however, we were not able to find sufficient published evidence to compare the rapid rises we found with other studies. There was a disproportionate number (15.5% of the 24-hour total) of rapid alkaline rises in the first hour after probe insertion, and this may reflect low-grade retching or gagging associated with reflux as the patient accommodated to the probe; alternatively the rapid alkaline rises in this first phase may be associated with the fasting state.

The second possible measure of DGR is prolonged nonacid pH in the stomach. An episode of raised intragastric pH unrelated to food ingestion has not generally been accepted as adequate evidence on its own of DGR. Verdu et al. [27] concluded from their study that periods of high antral pH are often due to swallowed saliva, secretion of bicarbonate by the antral mucosa, and sequestration of the probe in the gastric mucosa. Marshall et al. [26] added to this a combination of DGR and a reduction in meal-stimulated acid production occurring towards the end of the night. Verdu et al.'s conclusion of the unreliability of pH evidence on its own is possibly biased by their use of pH3 as the alkaline threshold [27]. pH >4 is more frequently used as the threshold for gastric alkaline shift [14, 26, 27]. Brown et al. [28] used a threshold pH of >4 and found a sensitivity of 84% for DGR compared with bile or amylase concentration. Fuchs et al. [29] developed a 16-variable computer programme which could reliably identify DGR using intragastric pH alone.

A normal pattern in other series is of nighttime alkaline peaks of pH4–pH7 lasting a few minutes to about an hour [11, 20], best demonstrated in the antrum. 46% of DGR episodes progress proximally to the fundus [14]. Barlow et al. [11] presumed these waves of alkalisation were due to DGR since there was no evidence of parietal cell rest in the proximal stomach during these episodes. Furthermore the rapid change and progression proximally in the stomach does not fit with other proposed explanations which include swallowed saliva, secretion of bicarbonate by the antral mucosa, or sequestration of the probe in the gastric mucosa. Björnsson and Abrahamsson [30], measuring antroduodenal pressures and gastric pH, concluded that nocturnal pH rises in the antrum are caused by duodenal retroperistalsis. The common occurrence in this study, of extensive nighttime alkaline plateaux including many prolonged episodes over pH7, and up to 9-hour duration supports the contention that there is a comparatively high level of DGR in this community. There is as yet no other data from Africa to indicate whether the DGR shown in this study is within normal limits for an African rural population.

All but one of the volunteers was positive for* H. pylori* IgG.

One volunteer was achlorhydric, and therefore her data was not included for analysis. The minimum pH obtained for the remaining volunteers was all 1.1 or less. Positive serology does not prove current infection; however, acid production may have been reduced by chronic* H. pylori* infection of the corpus. Tsai et al. [31] showed a rise in median pH from control levels of 1.4 ± 0.1 to pH 1.6 ± 0.3 in those with* H. pylori* infection. Furuta et al. [32] showed much greater reductions in acid production in subjects with active duodenal or gastric ulceration. All of the final 44 volunteers of the current study demonstrated the ability to create a low intragastric pH at some stage during the 24 hr period.

Most African studies report a high prevalence of* H. pylori* of 61–100% [33]. A study in Bloemfontein in South Africa [34] showed a rising prevalence during childhood, reaching 84.2% by the age of 15. At 97%, the prevalence rate in our volunteers is at the upper end of the spectrum.

Chebib et al. [35] found no correlation between the presence of* H. pylori* and enterogastric reflux; however,* H. pylori* was present more frequently in patients with positive enterogastric reflux. Nakagawara et al. [36] found that DGR facilitates the survival of* H. pylori* in the gastric stump after a gastrectomy. While there is no published evidence that* H. pylori* causes or facilitates DGR, the possibility must be considered that in our population the high prevalence of* H. pylori* infection is encouraged by a high level of DGR.

DGR is associated with raised intragastric pH [14, 28, 37]. There is a strong positive association of raised intragastric pH with nonacid gastroesophageal reflux [22, 29]. It is well established that pernicious anaemia and a postgastrectomy state are associated with reduced gastric acidity and also with squamous cancer of the oesophagus [38, 39].

Iijima et al. [40] demonstrated a risk for squamous cancer of the oesophagus of profound gastric hypochlorhydria after adjusting for the effect of gastric atrophy. An important question is whether a raised intragastric pH of a different nature compared to either achlorhydria or profound hypochlorhydria, and such as found in our volunteers, is a risk factor for squamous cancer of the oesophagus, and if so in what circumstances. The answer to this question is crucial for the community we have studied. It is also important to elucidate further the relation between enterogastric and gastroesophageal reflux.

## 5. Conclusion

Gastric alkalinisation is common in the sample population studied, at a higher pH than that reported in other studies of healthy subjects, and is sustained longer. Nighttime alkalinisation is particularly frequent. There is neither achlorhydria nor profound hypochlorhydria. This pattern is suggestive of a high level of duodenogastric reflux. The presence of this pattern in an area of very high risk for squamous cancer of the oesophagus requires further investigation.

## Figures and Tables

**Table 1:** 

24-hour and daytime pH results	
Mean 24-hour gastric pH	2.84
Lowest pH achieved (range of 0 to 4)	0.5 (0.2, 0.6)
Percentage of time gastric pH >4 (range of 0.4 to 65.9)	22.2 (13.9, 33.7)
Percentage of time supine pH >4 (range of 0 to 71)	26
Percentage of time gastric pH >7 (range of 0 to 37.4)	7.6 (0.6, 12.9)
Mean daytime gastric pH	2.67

Nighttime pH results (12 midnight to 6 am)	
Mean nighttime gastric pH	3.7
Nighttime minutes pH >4 (range of 0 to 359.6)	136.4 (22.8, 208.1)
Nighttime minutes pH >7 (range of 0 to 287.1)	79.3 (2.5, 122.7)

*Rapid alkaline rises *	
(Rises in less than 10 seconds from a baseline of pH2 or less, to a pH of 4 or more)	
Rapid alkaline rises in 24 hours (range of 0 to 390)	17 (10, 47)

**Table 2:** Other studies of healthy subjects.

Authors	Tutuian et al. [16]	Hartmann et al. [24]	Piccoli et al. [18]	Huang et al. [23]	Roman et al. [22]	This study
Female/male	20/16	0/20	19/17	0/74	6/6	26/18
Country	USA	Germany	Switzerland	Canada	France	South Africa
HP +ve	Not stated	7 of 20 +ve	Not stated	Not stated	Not stated	35 of 36
Mean/median 24 hr pH	2.05	1.4	1.3	2.39		2.84
Median daytime pH	2.8					2.67
Median nighttime pH	1.38					3.7
Nighttime pH >4	8.14%					9.40%
24 hr pH >4	20.03%		8.80%		14.50%	22.20%
Supine time pH >4					4.40%	26%
